# Toward a Unified Definition of Progression Independent of Relapse Activity in Multiple Sclerosis

**DOI:** 10.1212/WNL.0000000000213977

**Published:** 2025-09-22

**Authors:** Emilio Portaccio, Matteo Betti, Ermelinda De Meo, Luisa Pastò, Elio Prestipino, Alessandra Lugaresi, Eleonora Cocco, Giovanna De Luca, Francesco Patti, Valentina Liliana Adriana Maria Torri Clerici, Diana Ferraro, Vincenzo Brescia Morra, Giuseppe Salemi, Marika Vianello, Raffaella Cerqua, Matilde Inglese, Maria Barbara Pasanisi, Paola Perini, Silvia Romano, Carlo Pozzilli, Carla Tortorella, Alessia Di Sapio, Pietro Annovazzi, Marta Simone, Pietro Iaffaldano, Maria A. Rocca, Massimo Filippi, Maria Trojano, Maria Pia Amato, for the Italian Multiple Sclerosis and Related Disorders Register

**Affiliations:** 1 Department of NEUROFARBA, University of Florence, Italy;; 2 SOD Neurologia, Careggi University Hospital, Florence, Italy;; 3 UOSI Riabilitazione Sclerosi Multipla, IRCCS Istituto delle Scienze Neurologiche di Bologna, Italy;; 4 Dipartimento di Scienze Biomediche e Neuromotorie, Università di Bologna, Italy;; 5 Department of Medical Science and Public Health, Centro Sclerosi Multipla, University of Cagliari, Italy;; 6 Neurology Unit, Multiple Sclerosis Centre, SS. Annunziata University Hospital, Chieti, Italy;; 7 Department of Medical and Surgical Sciences and Advanced Technologies “G.F. Ingrassia”, University of Catania, Italy;; 8 Neuroimmunology Unit, Fondazione IRCCS Istituto Neurologico C. Besta, Milan, Italy;; 9 Dipartimento di Neuroscienze - Ospedale Civile di Baggiovara - Azienda Ospedaliero-Universitaria, Modena, Italy;; 10 Multiple Sclerosis Clinical Care and Research Center, Department of Neuroscience (NSRO), Federico II University, Naples, Italy;; 11 Department of Biomedicine, Neuroscience and Advanced Diagnostics, University of Palermo, Italy;; 12 Ca' Foncello Hospital, AULSS2, Neurology, Treviso, Italy;; 13 Clinica Neurologica - AOU delle Marche, Ancona, Italy;; 14 Department of Neuroscience, Rehabilitation, Ophthalmology, Genetics and Mother-Child Health (DINOGMI), University of Genoa, Italy;; 15 IRCCS Ospedale Policlinico San Martino, Genoa, Italy;; 16 Centro SM - Fondazione Don Carlo Gnocchi IRCCS, Milan, Italy;; 17 Centro regionale per la Sclerosi Multipla Regione Veneto, Clinica Neurologica, Azienda Ospedaliera, Università di Padova, Italy;; 18 Centro Neurologico Terapie Sperimentali - Università La Sapienza di Roma - AO S. Andrea, Rome, Italy;; 19 Centro SM - Policlinico S. Andrea - Università La Sapienza di Roma, Rome, Italy;; 20 Centro Sclerosi Multipla - AO S. Camillo Forlanini, Rome, Italy;; 21 Centro di riferimento Regionale SM (CRESM) - SCDO Neurologia - AOU San Luigi Gonzaga, Orbassano, Torino, Italy;; 22 Neurologia ad Indirizzo Neuroimmunologico - Centro Sclerosi Multipla - ASST della Valle Olona, Ospedale di Gallarate, Italy;; 23 Dipartimento di Medicina di Precisione e Rigenerativa e Area Jonica (DiMePre-J), University of Bari “Aldo Moro”, Italy;; 24 Department of Translational Biomedicine and Neurosciences, University of Bari “Aldo Moro”, Italy;; 25 Neuroimaging Research Unit, Division of Neuroscience, IRCCS San Raffaele Scientific Institute, Milan, Italy; Neurology Unit, IRCCS San Raffaele Scientific Institute, Milan, Italy; Vita-Salute San Raffaele University, Milan, Italy;; 26 Neuroimaging Research Unit, Division of Neuroscience, IRCCS San Raffaele Scientific Institute, Milan Italy; Neurology Unit, IRCCS San Raffaele Scientific Institute, Milan, Italy; Neurorehabilitation Unit, IRCCS San Raffaele Scientific Institute, Milan, Italy; Vita-Salute San Raffaele University, Milan, Italy;; 27 IRCCS Fondazione Don Carlo Gnocchi, Florence, Italy.

## Abstract

**Objectives:**

Progression independent of relapse activity (PIRA) is the main driver of disability accumulation in relapsing multiple sclerosis (MS). We tested various PIRA definitions against the risk of long-term disability.

**Methods:**

Patients with relapsing MS, first visit ≥January 1, 2000, ≥3 visits with Expanded Disability Status Scale (EDSS), and ≥5-year follow-up were extracted from the Italian MS and Related Disorders Register on September 29, 2023. Eighteen PIRA definitions were obtained combining fixed or roving baseline, 24-week, 48-week confirmed or sustained disability accrual, no relapses ≤90 days before/≤30 days after the event (90d), ≤180 days before/≤30 days after the event, or absence of relapses from baseline to confirmation score (ABS). Predictive performance against the reaching of EDSS = 6.0 was calculated.

**Results:**

A total of 30,203 patients were included. After a follow-up of 11.3 ± 4.3 years, PIRA ranged from 38.8% to 74.1%. EDSS = 6.0 was detected in 4,401 (15%) patients. Sensitivity of PIRA definitions against EDSS = 6.0 was higher using the 90d criterion (66.7%–69.4%), while the ABS criterion increased specificity (55.3%–62.2%).

**Discussion:**

The definition of PIRA combining roving baseline, no relapses 90 days before and 30 days after the event and 24-week confirmation achieved the best predictive value and feasibility, supporting its use in routine practice and research.

## Introduction

Multiple sclerosis (MS) research has increasingly focused on the relentless accumulation of disability independent of acute/subacute clinical manifestations of focal inflammatory activity, the progression independent of relapse activity (PIRA). Once believed to be confined to the progressive stages, PIRA is consistently observed in the early phases of relapsing MS,^1-4^ even in patients with pediatric onset.^5^ Across all phenotypes of the disease, PIRA emerged as the primary driver of disability accumulation.^3,4^

However, variations in PIRA definitions hindered the interpretability of key findings. Sources of heterogeneity include differing approaches to defining the 3 time points of disability accrual (DA): baseline, event, and confirmation scores. In addition, the duration of relapse-free intervals around the event score, used to distinguish PIRA from relapse-associated worsening, further contributes to inconsistencies.^6^ Recently, a harmonized definition of PIRA was proposed,^6^ but this consensus-based definition requires validation in real-world settings.

Because PIRA is the primary mechanism driving disability progression in MS, a reliable definition should obtain the highest predictive accuracy for long-term outcomes. In this analysis on the Italian MS and Related Disorders Register (MSARDR),^7^ we tested various PIRA definitions against the risk of reaching a robust disability milestone, the Expanded Disability Status Scale (EDSS)^8^ score of 6.0.

## Methods

This retrospective cohort study used prospectively acquired data extracted from the MSARDR on September 29, 2023 (detailed information on the Register is reported elsewhere^7^ and in eTable 1). In brief, the Register was approved by the Policlinico of Bari Ethics Committee and by the local committees in all centers. Written informed consent was obtained from all patients. This study followed the Strengthening the Reporting of Observational Studies in Epidemiology guidelines.

Patients with clinically isolated syndrome or relapsing-remitting course at the first visit, first visit ≥January 1, 2000, ≥3 visits with EDSS evaluation, and ≥5-year follow-up were included. We excluded patients with a primary or secondary progressive course at the first visit, enrolled in clinical trials and with missing data.

### PIRA Definitions

DA was defined as an EDSS score increase from baseline (≥1.5 if baseline EDSS = 0; ≥1.0 if baseline EDSS ≥1.0 and <5.5; ≥0.5 if baseline EDSS ≥5.5). Eighteen different definitions of PIRA were calculated by combining:

1. Fixed baseline (B_fix_: the first available visit in the database) or roving baseline (B_rov_).^6^ In particular, B_rov_ sets a new reference score every time the EDSS is lower than the previous measure and confirmed at the following visit. The EDSS is adjusted after relapses;
2. 24-week confirmed (24w), 48-week confirmed (48w) DA, or sustained (Sust) DA. When sustained, the EDSS score defining DA did not improve beyond the requirement for a significant EDSS score increase compared with the baseline (this applies to the 24 and 48-week periods, as well);
3. no relapses ≤90 days before and ≤30 days (90d) after and ≤180 days before and ≤30 days (180d) after the event and the confirmation scores or absence of relapses from baseline to confirmation score (ABS).

### Statistical Analysis

For each definition of PIRA, sensitivity, specificity, negative and positive predictive values (with 95% CI), and accuracy against the reaching of EDSS 6.0 were calculated. A sensitivity analysis stratifying subjects by age was performed.

All statistical analyses were performed with SPSS software, version 25.0 (SPSS Inc., College Chicago, IL). A 2-sided *p* value <0.05 was considered statistically significant.

### Data Availability

Anonymized data, not published in the article, will be shared on reasonable request from a qualified investigator.

## Results

**Figure 1:**
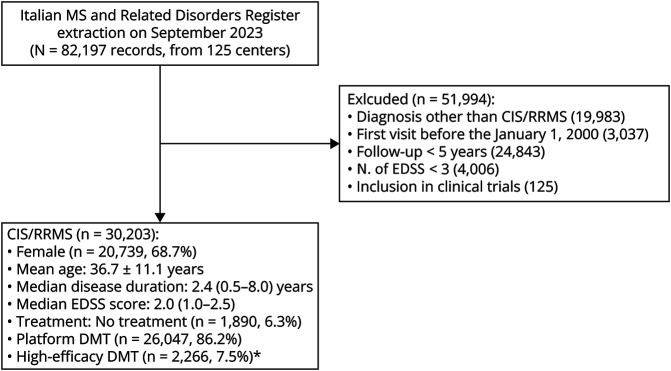
Flowchart of the Study Sample CIS = clinically isolated syndrome; DMT = disease-modifying treatment; EDSS = Expanded Disability Status Scale; MS = multiple sclerosis; RR = relapsing remitting. *Platform disease-modifying treatments: interferons, glatiramer acetate, dimethyl fumarate, teriflunomide, azathioprine. High-efficacy disease-modifying treatments: natalizumab, S1P-modulators, cladribine, antiCD20, alemtuzumab, cyclophosphamide, mitoxantrone.

By applying inclusion and exclusion criteria, we identified 30,203 patients (Figure 1).

**Figure 2:**
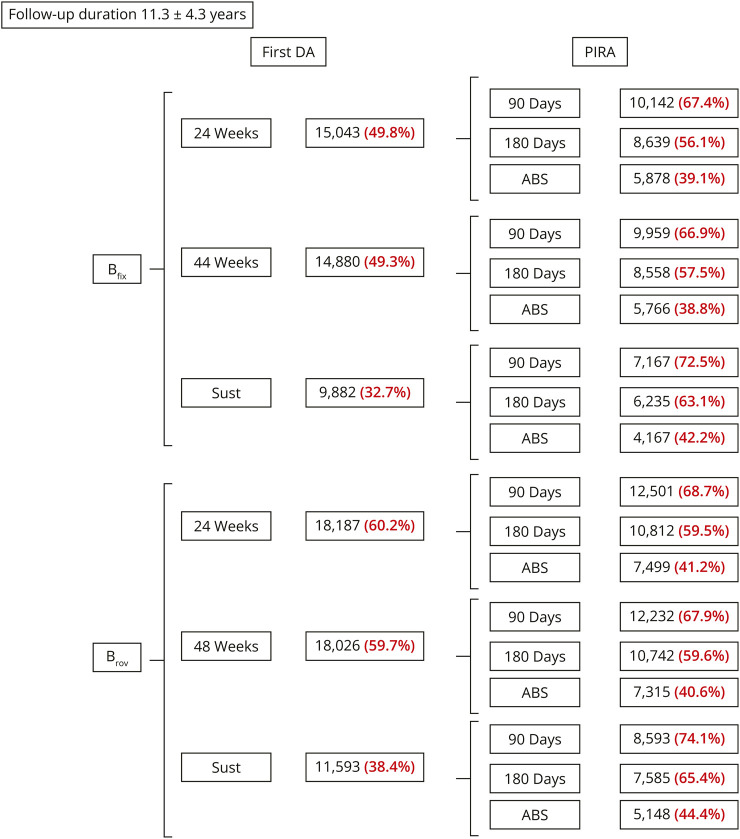
Proportion of Disability Accrual Events and Progression Independent of Relapse Activity According to Different Definitions 180d = no relapses <180 days before and <30 days after the event score; 24w = 24-week confirmation; 48w = 48-week confirmation; 90d = no relpases <90 days before and <30 days after the event score; ABS = no relapses from baseline to the confirmation score; Bfix = fixed baseline; Brov = roving baseline; DA = disability accrual; PIRA = progression independent of relapse activity; Sust = sustained disability accrual.

After a follow-up of 11.3 ± 4.3 years, using a fixed baseline, 15,043 (49.8%) 24w DA, 14,880 (49.3%) 48wDA, and 9,882 (32.7%) Sust DA were detected. Using a roving baseline, the corresponding values were 18,187 (60.2%) 24w DA, 18,026 (59.7%) 48w DA, and 11,593 (38.4%) Sust DA. Using the 90d criterion the proportion of PIRA ranged from 67.4% to 74.1%, from 56.1% to 65.4% with the 180d criterion, and from 39.1% to 44.4% with the ABS criterion (Figure 2).

At the end of the follow-up, EDSS 6.0 was detected in 4,401 (15%) of 29,410 patients with baseline EDSS ≤5.5. Predictive performance of the 18 different definitions of PIRA are depicted in Table 1. Overall, sensitivity was higher using the 90d criterion (66.7%–69.4%), while the ABS criterion increased specificity (55.3%–62.6%). The sensitivity analysis stratifying subjects by age confirmed the main findings (eTables 2–5).

**Table 1:** Predictive Performance of Different Definitions of Progression Independent of Relapse Activity Against the Risk of Reaching EDSS Score = 6.0 (4,401 [15%] of 29,410 Patients With Baseline EDSS ≤5.5)

Definition	Sensitivity	Specificity	NPV	PPV	Accuracy
B_fix_-24w-90d	66.7 (65.3–68.1)	32.9 (32.0–33.8)	69.6 (68.3–70.9)	30.0 (29.1–31.0)	43.1
B_fix_-24w-180d	54.9 (53.4–56.4)	42.3 (41.3–43.2)	68.5 (67.3–69.6)	29.1 (28.1–30.1)	46.1
B_fix_-24w-ABS	39.4 (38.0–40.9)	62.2 (61.2–63.1)	70.4 (69.4–71.3)	31.0 (29.8–32.3)	55.3
B_fix_-48w-90d	66.7 (65.3–68.1)	33.6 (32.7–34.5)	69.7 (68.4–71.0)	30.6 (29.7–31.5)	43.7
B_fix_-48w-180d	54.9 (53.4–56.4)	42.1 (41.1–43.1)	68.0 (66.9–69.2)	29.4 (28.4–30.4)	46.0
B_fix_-48w-ABS	39.4 (38.0–40.9)	62.6 (61.7–63.6)	70.2 (69.3–71.1)	31.7 (30.4–32.9)	55.6
B_fix_-Sust-90d	69.0 (67.5–70.5)	26.0 (24.8–27.1)	57.3 (55.4–59.1)	36.8 (35.7–37.9)	42.5
B_fix_-Sust-180d	57.8 (56.2–59.4)	34.5 (33.3–35.7)	56.7 (55.1–58.3)	35.6 (34.3–36.8)	43.5
B_fix_-Sust-ABS	41.0 (39.4–42.6)	58.4 (57.2–59.7)	61.3 (60.0–62.6)	38.1 (36.6–39.6)	51.7
B_rov_-24w-90d	66.7 (65.3–68.1)	31.2 (30.4–32.0)	73.8 (72.6–74.9)	24.4 (23.7–25.2)	40.1
B_rov_-24w-180d	54.9 (53.4–56.4)	39.7 (38.9–40.6)	72.6 (71.5–73.6)	23.3 (22.5–24.1)	43.5
B_rov_-24w-ABS	39.4 (38.0–40.9)	59.2 (58.3–60.0)	74.6 (73.7–75.4)	24.3 (23.4–25.3)	54.2
B_rov_-48w-90d	66.7 (65.3–68.1)	32.3 (31.5–33.1)	74.2 (73.1–75.3)	25.0 (24.2–25.8)	41.0
B_rov_-48w-180d	54.9 (53.4–56.4)	39.6 (38.8–40.5)	72.3 (71.2–73.3)	23.5 (22.7–24.3)	43.5
B_rov_-48w-ABS	39.4 (38.0–40.9)	60.0 (59.2–60.9)	74.6 (73.8–75.4)	25.0 (24.0–26.0)	54.8
B_rov_-Sust-90d	69.4 (67.9–70.9)	24.3 (23.3–25.2)	62.1 (60.4–63.9)	30.7 (29.7–31.7)	39.0
B_rov_-Sust-180d	58.4 (56.8–60.0)	32.0 (30.9–33.0)	61.4 (59.8–62.9)	29.3 (28.3–30.4)	40.6
B_rov_-Sust-ABS	41.3 (39.7–42.9)	55.3 (54.2–56.5)	66.1 (64.9–67.3)	30.9 (29.6–32.2)	50.8

Abbreviations: 180d = no relapses ≤180 days before and ≤30 days after the event score; 24w = 24-week confirmation; 48w = 48-week confirmation; 90d = no relapses ≤90 days before and ≤30 days after the event score; ABS = no relapses from baseline to the confirmation score; B_fix_ = fixed baseline; B_rov_ = roving baseline; EDSS = Expanded Disability Status Scale; NPV = negative predictive value; PPV = positive predictive value; Sust = sustained disability accrual.

## Discussion

In this real-world cohort study, the definition of PIRA combining roving baseline EDSS, no relapses 90 days before and 30 days after the event and the confirmation scores, and 24-week confirmed DA achieved the best balance of sensitivity, negative predictive value, and feasibility in predicting the long-term risk of EDSS 6.0.

Compared with fixed baseline, roving baseline increased detection of disability accumulation by 17%–19%, aligning with previous studies,^9,10^ which highlights the need to account for disability improvements prior to worsening. Such improvements could be due to recovery from earlier relapses^11^ or to EDSS variability from inter-rater differences and symptom fluctuations.

A relapse-free period of 90 days before and 30 days after the event offered greater sensitivity and negative predictive value compared with shorter intervals, such as those applied in clinical trials. The adoption of a standardized definition of relapse is crucial. Relapses are usually defined as acute/subacute episodes of worsening symptoms lasting ≥24 hours, occurring ≥30 days after a prior episode, and without fever or illness.^6^ However, this definition may miss milder episodes, particularly with infrequent clinical visits, affecting the DA phenotype definition. While stringent criteria excluding relapses from baseline to confirmation increase specificity, they reduce sensitivity, making the definition dependent on the study's purpose. Narrower time intervals, commonly used in the literature,^6,12^ show PIRA as the primary driver of disability accumulation in relapsing MS (∼67%–74%), but proportions fall below 50% when broader intervals are applied. Notably, the proportion of PIRA events could be further reduced when other focal inflammatory parameters, such as those from MRI, are included.

Overall, the choice of relapse-free intervals should reflect study goals. For prognostic purposes, narrower intervals (e.g., 90 days before and 30 days after) optimize sensitivity. By contrast, longer intervals enhance specificity for “true” PIRA, particularly in research contexts.

Definitions requiring sustained confirmation of DA offer the highest sensitivity. This finding is in line with a previous observation linking sustained PIRA to a higher risk of transition to secondary progressive MS.^13^ However, this definition is impractical in clinical settings. A 24-week confirmation interval is a more feasible alternative with minimal sensitivity loss (2%–3%).

This study has limitations. It focused on the first DA, limiting its applicability to longitudinal cohorts. However, the analysis of repeated PIRA would carry several issues, such as baseline definition, overlap of confirmation intervals, and interpretation of different PIRA types in the same individual. The DA was based on the EDSS alone, potentially underestimating PIRA by excluding measures like the Timed 25-Foot Walk and 9-Hole Peg Tests or progression of cognitive impairment.^1,14^ The absence of MRI data further limits the ability to rule out subclinical inflammatory contributions. Notably, PIRA definitions had a relatively poor performance in predicting EDSS 6.0, likely because of the low frequency of the outcome (15%). Moreover, the whole cohort was used for all definitions and a validation in an independent sample would be relevant. Nonetheless, the proposed definition of PIRA as any 24-week confirmed DA, with roving baseline and no relapses 90 days before and 30 days after the event and confirmation scores, balances sensitivity, feasibility, and predictive value, supporting its use in clinical trials, real-world research, and routine practice to identify treatments for relapse-independent disability progression.

## Appendix Coinvestigators

**Table:**

Coinvestigators are listed at Neurology.org.
